# A53T α-Synuclein Expression is Associated with Altered Dopaminergic-Like Differentiation and Reduced DNA Topoisomerase IIβ Levels in an In Vitro Model of Parkinson’s Disease

**DOI:** 10.1007/s12035-026-05881-1

**Published:** 2026-05-11

**Authors:** Aslınur Selim, Timuçin Avşar, Yeşim Neğiş, Sevim Işık

**Affiliations:** 1 Graduate School of Sciences, Üsküdar University, Istanbul, Turkey; 2 Department of Molecular Biology and Genetics, Faculty of Science and Engineering, Üsküdar University, Istanbul, Turkey; 3 Stem Cell Research and Application Center (ÜSKÖKMER), Üsküdar University, Istanbul, Turkey; 4 Department of Medical Biology, Faculty of Medicine, Bahçeşehir University, Istanbul, Turkey; 5 Department of Medical Biochemistry, School of Medicine, Bahçeşehir University, Istanbul, Turkey

**Keywords:** DNA topoisomerase IIß, Parkinson’s disease, A53T mutant α-synuclein, SH-SY5Y cells, Nurr1

## Abstract

The study explores the interaction between topoisomerase IIβ (topo IIβ) and α-synuclein aggregation in Parkinson’s disease (PD) using an in vitro model overexpressing A53T mutant α-synuclein, revealing key pathological features. The overexpression of A53T mutant α-synuclein was associated with impaired neuronal differentiation, accompanied by delayed neurite outgrowth and reduced levels of MAP2 and NF-L. Transcriptomic study during differentiation revealed substantial gene expression changes, with 57 of 84 PD-associated genes exhibiting differential regulation on days 8 and 12. Dopaminergic markers like TH, BDNF, NR4A2 (Nurr1), PINK1, and SYNGR3 were notably upregulated in differentiated cells, whereas A53T mutant α-synuclein overexpression was associated with significant downregulation of 22 genes associated with PD, including TH, NR4A2, BDNF, and PINK1, suggesting alterations in neuroprotective signaling pathways. Topo IIβ expression diminished during differentiation in the presence of pathogenic α-synuclein and was associated with changes in the proposed topo IIβ-Nurr1 regulatory axis potentially contributing to early neurodevelopmental abnormalities and dopaminergic vulnerability observed in PD.

**Supplementary Information:**

The online version contains supplementary material available at 10.1007/s12035-026-05881-1.

## Introduction

Parkinson’s disease (PD) is a progressive neurodegenerative disease characterized by the death of dopaminergic neurons in the substantia nigra pars compacta (SNpc) [1] and Lewy neurites (LNs), in neuronal axons [2, 3] linked to neuronal death. Among the genetic contributors, mutations in the SNCA gene play a central role [4], with particularly the A53T point mutation being the earliest identified cause of autosomal dominant PD [5]. A53T accelerates α-synuclein (α-Syn) aggregation and increases its susceptibility to pathogenic phosphorylation, making it a widely used in vitro model for investigating early α-Syn-related molecular events [6, 7]. Additionally, functional relationships have been demonstrated between SNCA and genes such as LRRK2, PINK1, and Parkin, which are associated with PD pathology [8]. In addition to protein aggregation, growing evidence indicates that transcriptional dysregulation also contributes to PD. One of these molecular relationships involves the enzyme DNA topoisomerase IIβ (topo IIβ), which regulates DNA topology during transcription [9, 10] and has emerged as a critical factor in neural differentiation [11]. Additionally, topo IIβ null mice have a prenatal death phenotype with failed axon growth [12], highlighting its role in neural differentiation and brain development. In addition, in vitro studies have shown that topo IIβ inhibitors prevent neurite growth and growth cone formation [13] and similarly topo IIβ-specific siRNAs downregulate genes related to neuronal development while increasing those related to Alzheimer’s disease (AD) and PD [14]. Also, suppressing TOP2B activity in neural-differentiated human mesenchymal stem cells hinders neural differentiation and axonogenesis, while overexpression results in longer axons [15].

On the other hand, a significant interaction has been found between the transcription factor Nurr1, which has a role in development and maintenance of midbrain dopamine neurons [16], and topo IIβ. Reports indicate that topo IIβ expression is diminished in Nurr1 knockout mice [17], and Nurr1 levels are similarly decreased when topo IIβ is silenced [15]. These findings indicate a coordinated relationship between topo IIβ and Nurr1 in dopaminergic neurons [15], [17]. 

Emerging evidence also implicates the role of topo IIβ in neurodegenerative diseases, specifically in models of Alzheimer’s disease (AD) [18] and PD [19, 20]. The MPP^+^-induced PD model exhibited a decrease in topo IIβ expression and a concomitant reduction in tyrosine hydroxylase (TH) levels [15]. This suggests that topo IIβ may be directly linked to dopaminergic functions.

A recent preclinical study using an age-dependent transgenic mouse model of PD found a significant reduction in DNA topo IIβ and Nurr1 expression levels in the transgenic mice, particularly in the early stage. These findings suggest that decreased topo IIβ and Nurr1 levels, along with increased neuroinflammation, may contribute to the early stage of PD, suggesting topo IIβ as a potential intervention target [19].

In this context, we investigated the association between A53T mutant α-Syn gene overexpression and the expression of topo IIβ and 84 different PD-associated genes, including Nurr1, using neuronally differentiated SH-SY5Y cells as an in vitro PD-related model. This cell model is frequently preferred for investigating PD pathophysiology due to its catecholaminergic properties and neuronal differentiation capacity in PD [21, 22]. Following A53T transfection, we observed α-Syn accumulation accompanied by reduced expression of the dopaminergic marker TH, while topo IIβ and Nurr1 expression levels were also decreased. Our findings provide new insights into the molecular alterations associated with A53T mutant α-Syn expression and contribute to a better understanding of the potential involvement of topo IIβ in PD-related cellular processes.

## Materials and Method

### SH-SY5Y Culture

SH-SY5Y cells were cultivated in Dulbecco’s modified Eagle’s medium (DMEM) with 15% fetal bovine serum (FBS) and incubated at 37 ℃ with 5% CO2. Medium refreshment was done every 48 h. When the cells reached a confluency of 70–80%, they were detached using 0.25% Trypsin/EDTA solution and then seeded in a cell-culture flask at a density of 4 × 104 cells/cm^2^. Cells were subcultured every 7 days regularly.

### In Vitro Parkinson’s Disease Model Through Transfection of SH-SY5Y Cells with A53T Mutant α-Syn Plasmid

EGFP-α-Syn-A53T (Addgene) plasmid isolation was carried out with ZymoPURETM II Plasmid Maxiprep kit (Zymo Research, D4203) following the recommended instructions. The concentration of the isolated plasmid DNA was determined using an LVis plate and a multimode microplate reader. The SH-SY5Y cell line was cultured in a flask with suitable conditions (5% CO2 at 37 °C in DMEM supplemented with 15% FBS). Once the cell density reached 70%, the cells were transfected with the A53T mutant α-Syn plasmid using Lipofectamine P3000 reagent (Invitrogen) diluted with Opti-MEM (Gibco). The prepared mixture was incubated for 10 to 15 min at room temperature (RT) for the DNA and Lipofectamine reagents to form a complex. This complex was added onto the seeded cells in a drop-by-drop manner. Cells were incubated for 36h, then sorted by fluorescence-activated cell sorting (FACS, BD InfluxTM Cell Sorter) in order to homogenize the cell population to be only transfected cells. GFP-α-Syn(+) SH-SY5Y cells were then seeded into well plates to induce neural differentiation.

### Neural Differentiation of SH-SY5Y Cells with RA and BDNF

**Fig. 1:**
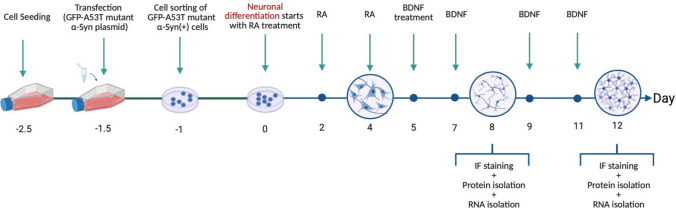
The chronological sequence of the experimental procedure. The figure illustrates the steps involved in transfecting SH-SY5Y cells with EGFP-α-Syn-A53T plasmid and differentiating SH-SY5Y cells with RA on days 0, 2, and 4, followed by BDNF on days 5, 7, 9, and 11. The immunofluorescence staining experiment was performed on days 8 and 12. The process of isolating protein and RNA for Western blot analysis and RT-qPCR profiler assay, respectively, was performed on days 8 and 12

Prior to cell seeding, well plates were coated with fibronectin to initiate neuronal differentiation. Neuronal differentiation was induced 24 h later using differentiation medium 1 (day 0), which included 10 µM concentration of retinoic acid (RA, Sigma) in F12 medium (15% FBS and 1% Pen-Strep). RA medium was renewed every 48 h up to day 5 of differentiation. On the fifth day, differentiation medium 2 which consisted of brain-derived neurotrophic factor (BDNF, Abcam) in F12 medium w/o FBS was applied, and it was renewed every 48 h including the 11th day. On day 12, the neuronal differentiation process was completed. Neuronal differentiation time plan is explained in Fig. 1.

SH-SY5Y cells were seeded according to the experimental groups (Table 1) to be analyzed at the protein level (Western blot and immunofluorescence staining) and mRNA level (Human Parkinson’s Disease RT^2^ Profiler PCR Array). The experimental timeline is displayed in Fig. 1.

**Table 1:** Abbreviations

Abbreviations		Meaning
Undiff control Diff control α-Syn(A53T(+))/Diff	Experimental groups	SH-SY5Y (undifferentiated) Neural differentiated SH-SY5Y A53T mutant α-Syn overexpressed SH-SY5Y induced to neural differentiation
α-Syn A53T(+)		A53T mutant α-Syn overexpression

### Immunofluorescence Staining of SH-SY5Y Cells

Immunofluorescence staining (IF) was performed in 24-well plates, at certain days of ongoing neuronal development. Initially, cells were fixed with 4% PFA/PBS buffer, and following a 1× PBS wash, permeabilized with TZN buffer (10 mM pH 7.5 Tris-HCl, 0.5% Nonidet P-40, 0.2 mM ZnCl2). Cells were incubated with the blocking solution (0.3% PBS/Triton X (PBS-Tx), 5% normal goat serum (NGS), and 5% normal horse serum (NHS) at RT for a duration of 40 min. Then, cells were exposed to primary antibodies that were diluted in PBS-Tx and 3% NHS, targeting specific proteins, and incubated overnight at 4 °C. Cells were washed and incubated with Alexa Fluor-labelled anti-mouse or anti-rabbit secondary antibodies for 1 h. In order to stain the nucleus, cells were incubated with 1:5000 × DAPI solution for 5 min. Following the washing steps, ProLong Glass Antifade Mountant (Invitrogen) was applied onto the cells, and a coverslip was placed over them. Staining of the cells was observed under the fluorescent microscope (Carl Zeiss AG, Oberkochen, Germany). Images were sampled as *n* = 5 and quantification of neuronal branching of neuronal markers was analyzed using WimNeuron assay. Primary antibodies against topo IIβ (1:100, BD), Nurr1 (1:100, Invitrogen), tyrosine hydroxylase (TH, 1:100, Abcam), phosphorylated-α-Syn (p-α-Syn, 1:100, Cell Signaling), neurofilament light chain (NF-L, 1:50, Cell Signaling), and microtubule-associated protein 2 (MAP2, 1:100, Cell Signaling) were applied at the specified ratios. Secondary antibodies, GAM-IgG-Alexa Fluor 594 (1:100, Invitrogen) and GAR-IgG-Alexa Fluor 594 (1:100, Invitrogen), were used.

### Western Blot Analysis

Proteins were isolated from SH-SY5Y cells seeded in 6-well plates and then subjected to Western blot analysis. Lysis of cells was performed by adding RIPA buffer (1% protease inhibitor, 1% SDS) to the cells and scraping them from the bottom of the plate. The samples were subjected to two rounds of homogenization using the MagNA Lyser (Roche) at 6000 rpm for a duration of 15 s. Subsequently, they were heated to 95 °C and boiled for 2 min. The concentration of samples was measured by Rapid Gold BCA Protein Test Kit (Invitrogen, A53225). The obtained samples from different experimental sets (*n* = 3) were pooled to obtain sufficient material for analysis. Eleven micrograms of samples was loaded into 5% and 10% polyacrylamide gel according to the molecular weight of the targeted proteins. Once the gel electrophoresis process was finished, blotting of the proteins to a polyvinylidene difluoride (PVDF) membrane was carried out. The membrane was incubated with the blocking solution (5% skimmed milk in NewTBS (1 M Tris-HCl (pH 7.5) + 5 M NaCl in dH2O) + 0.1% Tween 20) at RT for 1 h. This was followed by incubation with primary antibodies overnight and for an additional 2 h at RT. The primary antibodies used were topo IIβ (1:1000), TH (1:2000), Nurr1 (1:1000), p-α-Syn (1:1000), actin (1:7500, Boster), and nucleolin (1:1000, Novus Biologicals) which were diluted in an antibody solution containing 5% skim milk in New TBS + 1% Tween20. The membrane was washed with TTBS (a mixture of Tris-buffered saline and 0.05% Tween20) for 3 times and was incubated with goat anti-mouse (GAM) secondary antibody, HRP conjugate (1:1000, Abcam), and goat anti-rabbit (GAR) secondary antibody, HRP conjugate (1:1000, Abcam) at room temperature for 1 h. The membrane was treated with the mixed substrates of enhanced chemiluminescence (ECL), and digital screening was performed using LI-COR.

### Human Parkinson’s Disease RT^2^ Profiler PCR Array

The isolation of total RNA from SH-SY5Y cells planted in 6-well plates was performed using the RNeasy Mini Kit (Qiagen, #74904) in accordance with the instructions of the manufacturer. Subsequently, the obtained RNA samples were reverse-transcribed using the first-strand kit of RT2-Profiler PCR Array for PD (Qiagen). The samples were then combined with SYBR Green PCR mixture and were submitted to RT2-Profiler PCR Array analysis, which consisted of 84 genes (SLC6A3, CASP1, FGF13, SV2B, CASP7, NFASC, VAMP1, PSEN2, SYT11, KCNJ6, ATXN2, ALDH1A1, TPBG, CUL2, HSPA4, MAPT, UBE2L3, CADPS, PARK2, CASP3, CDC42, UBA1, BDNF, NSG1, TH, DDC, SNCA, SEPT5, CXXC1, YWHAZ, FBX09, BASP1, SYNGR3, PINK1, GBE1, SLC25A4, APC, UCHL1, GPR37, SLIT1, DRD2, SYT1, STUB1, NR4A2, APP, OPA1, UBE21, RGS4, FN1, CHGB, UBB, PRDX2, NTRK2, SKP1, MAPK9, NSF, SPEN, SLC18A2, HTR2A, S100B, PAN2, DLK1, PTEN, CDC27, EGLN1, ATP2B2, NEFL, CASP8, CASP9, UBE2K, LRRK2, NCOA1, GABBR2, RTN1, CDH8, TCF7L2, VDAC3, SRSF7, ATXN3, NRXN3, PARK7, PPID, GRIA, USP34) related to PD.

### Statistical Analysis

Statistical analysis was carried out using GraphPad Prism 9. Data are shown as the mean ± standard deviation (SD) of three separate studies. One-way ANOVA and two-way ANOVA were used to assess the differences among the experimental groups. The statistical significance of differences was established at *p < 0.05, **p < 0.01, ***p < 0.001, ****p < 0.0001.

## Results

### While SH-SY5Y Cells Differentiated Effectively with RA and BDNF, A53T Mutant α-Synuclein Overexpression Delayed Neural Differentiation and Inhibited Neurite Outgrowth

**Fig. 2:**
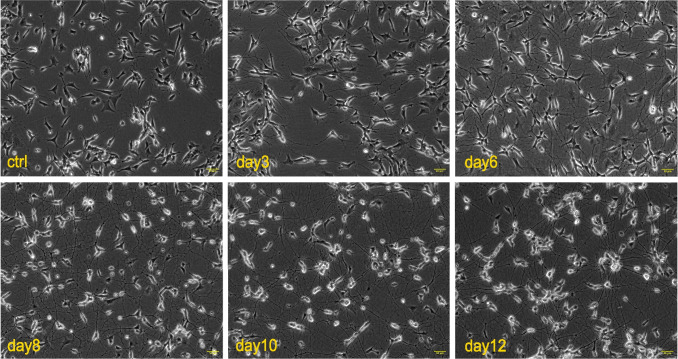
SH-SY5Y cells were induced to neuronal differentiation with RA and BDNF. Representative images show the morphological changes observed in SH-SY5Y cells that were differentiated into neuronal cells by treating the cells with RA and BDNF, respectively. The cells exhibited extended neurites and a more neuron-like morphology starting from day 8, characteristic of successful differentiation. The images were taken under phase contrast microscope

Neuronal differentiation of SH-SY5Y cells was performed according to the protocol described previously [20]. Cellular morphology was monitored daily throughout the differentiation process using phase-contrast microscopy. During the early stages of differentiation, following the initiation of RA treatment, cells exhibited increased spreading and progressively developed neurite-like extensions. Upon exposure to BDNF beginning on day 5 of differentiation, a marked reduction in cell body size was observed, accompanied by an increase in neurite number. Neurite elongation became more pronounced as differentiation progressed (Fig. 2).

**Fig. 3:**
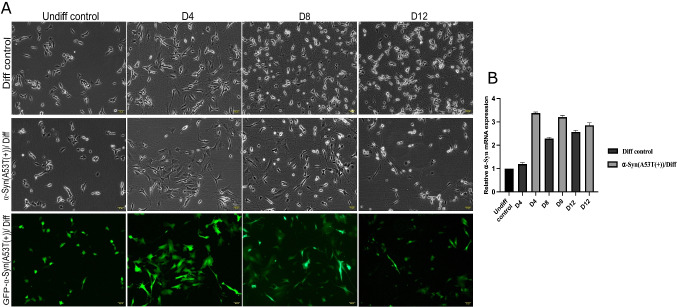
The overexpression of GFP-α-Syn/A53T had a role in inhibiting SH-SY5Y cells from undergoing neuronal differentiation. **A** Representative images of SH-SY5Y cells display the morphological difference between Diff control and α-Syn(A53T(+))/Diff groups on day 4, 8 and 12 of neuronal differentiation. **B** Relative α-Syn mRNA expression displaying A53T mutant α-Syn transfection efficiency. The Diff control group exhibited gradually increased neuronal network formation. On the other hand, in the α-Syn(A53T(+))/Diff group, there were still some cells that did not undergo neuronal differentiation even though cells started to extend their neurites in the early days of differentiation

Previous studies have demonstrated that α-Syn A53T(+) induces toxicity in dopaminergic neurons [23]. SH-SY5Y cells are widely used as an in vitro model for PD research [22]. Accordingly, SH-SY5Y cells were transfected with an A53T mutant α-Syn-encoding plasmid and subsequently incubated to model A53T mutant α-Syn-mediated cellular toxicity. Highest GFP fluorescence, indicating A53T α-Syn expression, was observed 36 h post-transfection. Fluorescence-activated cell sorting (FACS) revealed a transfection efficiency of approximately 30% in GFP-A53T-α-Syn-positive SH-SY5Y cells. Additionally, α-Syn overexpression was validated at mRNA level using RT-qPCR (Fig. 3B). Neuronal differentiation of the sorted cells was initiated immediately following FACS.

Morphological comparisons between the Diff control and the α-Syn(A53T(+))/Diff are shown in Fig. 3. At the earliest time point, cell numbers differed by approximately 20%; however, this disparity became more pronounced over time, reaching nearly 66% by the end of the differentiation period suggesting that the observed changes are unlikely to be solely attributable to FACS-related technical factors. In addition, a control experiment in which the initial seeding density was varied by ± 20% is presented (Supplementary Fig. 1) to evaluate the potential impact of cell number on neuronal differentiation. These findings indicate that the observed alteration in neuronal differentiation may be associated with α-Syn A53T(+) and is unlikely to be solely explained by differences in initial cell number. α-Syn(A53T(+))/Diff exhibited delayed progression into neuronal differentiation, with reduced neurite formation compared with the Diff control. In contrast to Diff control, neuronal branching was significantly impaired, and an increased rate of cell death was observed in α-Syn(A53T(+))/Diff.

**Fig. 4:**
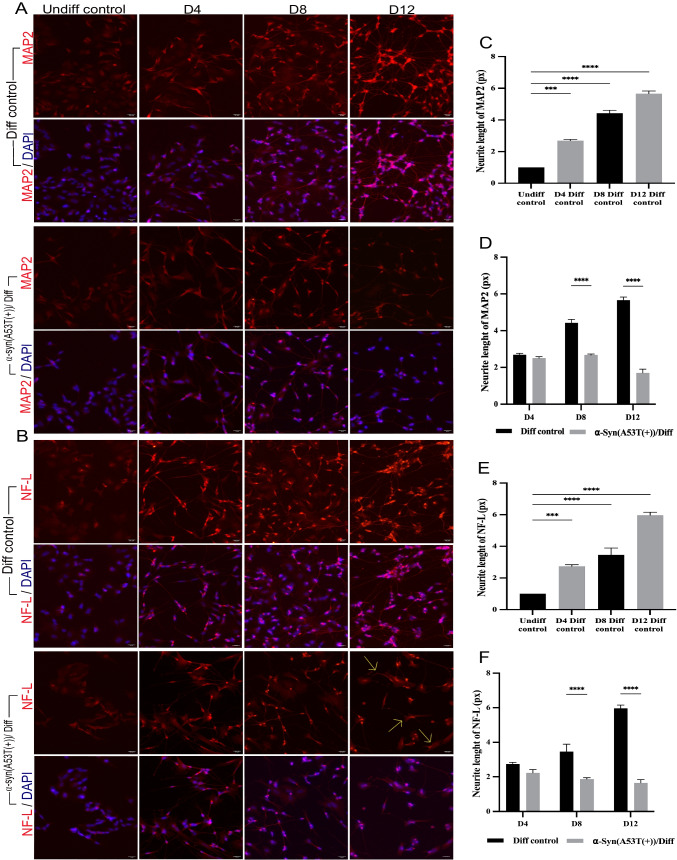
α-Syn A53T(+) delayed neural differentiation and inhibited neurite outgrowth in SH-SY5Y cells. Immunofluorescence staining of **A** MAP2 and **B** NF-L in Undiff control, Diff control and α-Syn(A53T(+))/Diff on different days of neuronal differentiation. The images were taken under fluorescent microscope (20×). Neurite length analysis of **C** and **D** MAP2 and **E** and** F** NF-L in the same experimental groups on different days of neuronal differentiation. Error bars represent the means ± standard deviation; *n* = 3 samples in triplicate; ***p < 0.001, ****p < 0.0001. *Significance levels between groups. Neurite lengths of MAP2 and NF-L in IF staining were measured using WimTube formation assay

To further evaluate the effect of α-Syn A53T(+) on the neuronal differentiation of SH-SY5Y cells, IF staining for the neuronal markers MAP2 and NF-L was performed on days 4, 8, and 12 of neuronal differentiation. The fluorescent images of the Diff control and the α-Syn(A53T(+))/Diff group stained with neuronal markers are shown in Fig. 4.

In the Diff control group, the expression levels of both MAP2 and NF-L gradually increased over the course of neuronal differentiation. Neuronal network formation was clearly observed by day 12, consistent with the progression of neuronal differentiation. In contrast, MAP2 expression in the α-Syn(A53T(+))/Diff group decreased as cells were induced toward neuronal differentiation, indicating a negative impact of α-Syn A53T(+) on neuronal differentiation. The NF-L marker displayed a similar expression pattern to that of MAP2 in the α-Syn(A53T(+))/Diff group. On day 12 of neuronal differentiation, it was also observed that a subset of cells did not undergo neuronal differentiation (indicated by the yellow arrow in Fig. 4). Neurite lengths of MAP2 and NF-L in IF staining were quantified using the WimTube formation assay. Neuronal differentiation efficiency was quantified as 75% in the Diff control group. Consistent with expression levels, both MAP2 and NF-L neuronal biomarkers exhibited the highest neurite length in the Diff control group, whereas the shortest neurite length was observed in the α-Syn(A53T(+))/Diff group on day 12 of differentiation. By day 12, neurite outgrowth in the α-Syn(A53T(+))/Diff group was reduced to nearly one-fourth of that observed in the Diff control group (Fig. 4).

### A53T Mutant α-Syn Overexpression in SH-SY5Y Cells Resulted in the Suppression of Topo IIβ

**Fig. 5:**
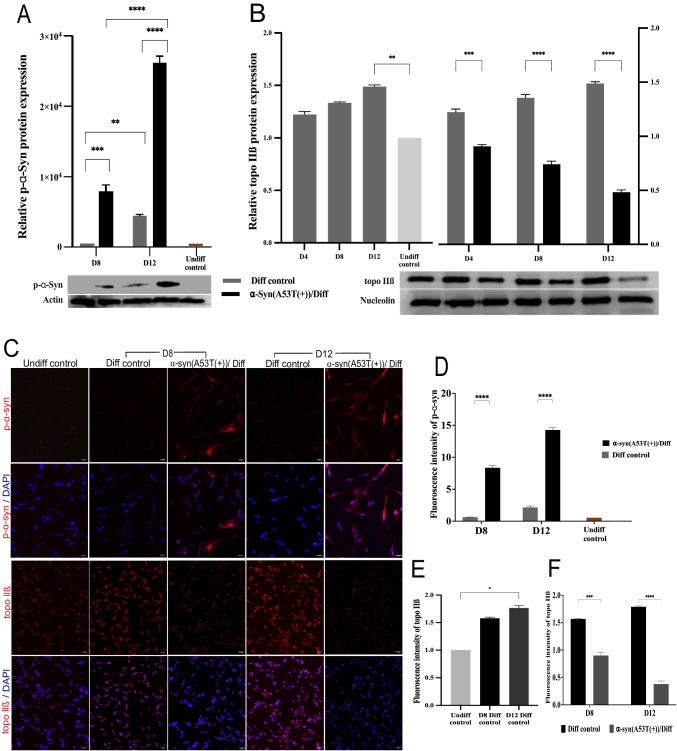
α-Syn A53T(+) increased the p-α-Syn expression, and a corresponding decrease in topo IIβ expression was observed in SH-SY5Y cells. Western blot analysis of **A** p-α-Syn, and **B** topo IIβ and IF staining of **C** p-α-Syn and topo IIβ in the Undiff control, Diff control and α-Syn(A53T(+))/Diff on different days of neuronal differentiation. The images were taken under a fluorescent microscope (20×). Neurite length analysis of **D** p-α-Syn and **E** and **F** topo IIβ from the IF staining images. Error bars represent the means ± standard deviation; for Western blot, *n* = 3 samples in triplicate; *p < 0.05, **p < 0.01, ***p < 0.001, ****p < 0.0001. *Significance levels between groups

To investigate the effect of α-Syn A53T(+) on topo IIβ, a Western blot analysis was performed across the experimental groups (Fig. 5). Previous research has recognized that the phosphorylation of α-Syn is an indication of a pathological α-Syn modification and is considered a pathogenic marker intimately linked to PD[24]. Our results showed that the expression of p-α-Syn was the highest (*p* < 0.0001) in the α-Syn(A53T(+))/Diff group compared with the Undiff control on day 12 of differentiation. The elevated p-α-Syn expression observed in the α-Syn(A53T(+))/Diff group is consistent with the previous findings and with the outcomes of the present study, supporting its potential relevance as a marker associated with PD. The correlation between p-α-Syn (Fig. 5A) and topo IIβ (Fig. 5B) at the protein level is shown. To assess the effect of neuronal differentiation on topo IIβ expression, a preliminary analysis was conducted. This analysis revealed a significant increase (*p* < 0.01) in the expression of topo IIβ on day 12 in the Diff control group compared with the Undiff control group, further supporting the role of topo IIβ in neuronal differentiation. Analysis of topo IIβ expression in the α-Syn(A53T(+))/Diff group revealed a gradual decrease as differentiation progressed, in contrast to the Diff control group. The difference in topo IIβ expression between the α-Syn(A53T(+))/Diff group and the Diff control group was found to be significant (*p* < 0.001) as early as day 4 of differentiation. Moreover, on day 12, which corresponded to the peak of p-α-Syn expression in the α-Syn(A53T(+))/Diff group, topo IIβ expression was significantly reduced, reaching the lowest observed level (*p* < 0.0001). These findings further support an association between α-Syn A53T(+) in SH-SY5Y cells and reduced topo IIβ expression.

Furthermore, to clarify the Western blot results, IF staining was performed across the experimental groups (Fig. 5C), and the corresponding fluorescent intensities were analyzed (Fig. 5D, E, and F). Compared with the Undiff control and Diff control groups, p-α-Syn staining was more prominent on days 8 and 12 of differentiation in the α-Syn(A53T(+))/Diff group.

Topo IIβ was specifically localized to the cell nucleus, consistent with its role in DNA metabolism [25]. As differentiation progressed, the topo IIβ signal in the Diff control group gradually increased, reaching a significant elevation (*p* < 0.05) on day 12. In contrast, when compared with the Diff control group, weaker topo IIβ signal intensities were observed in the α-Syn(A53T(+))/Diff group on days 8 and 12 of neuronal differentiation (*p* < 0.001 and *p* < 0.0001, respectively). This reduction was notable given that p-α-Syn exhibited stronger signal intensity in the same group at the corresponding time points. Consequently, these findings were consistent with the Western blot analysis. Taken together, the protein-level results, including the decrease in topo IIβ expression and the observed delay in neuronal development, indicate that α-Syn A53T(+) is associated with a significant alteration in topo IIβ.

### Topo IIβ and Nurr1 Show Reduced Expression in Response to A53T Mutant α-Syn Overexpression in PD-Related Genes

**Fig. 6:**
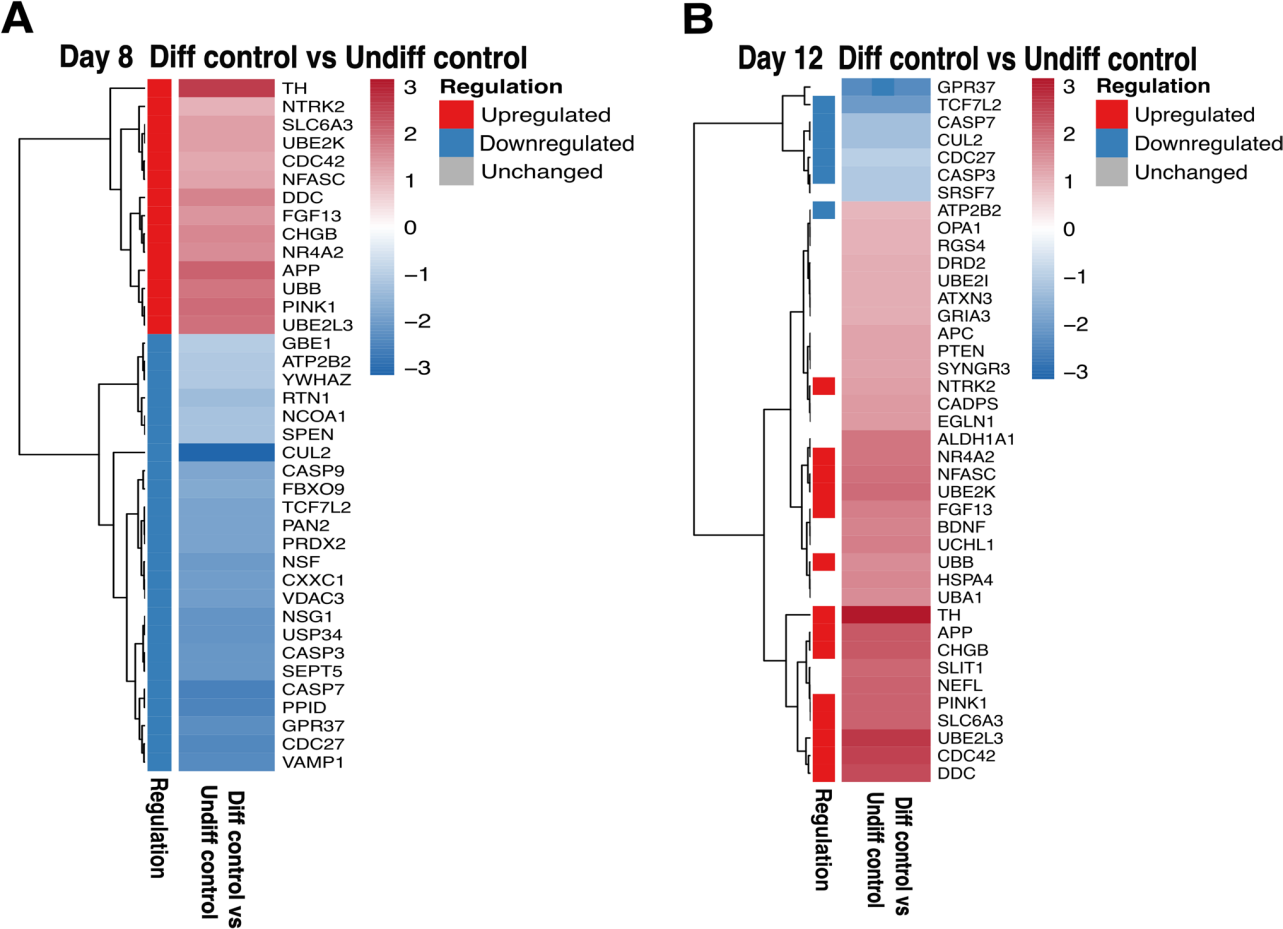
Heat maps of Human Parkinson’s Disease RT^2^ Profiler PCR Array data demonstrating the difference in PD-related gene expressions of **A** Day 8 Diff control and **B** Day 12 Diff control groups normalized to the Undiff control group. Genes with fold regulation (FR) > 2 were found to be upregulated whereas those with FR < 1 were shown to be downregulated. The test group: Diff control and the control group: Undiff control. The genes displayed here are listed as: ALDH1A1, APC, APP, ATP2B2, ATXN3, BDNF, CADPS, CASP3, CASP7, CASP9, CDC27, CDC42, CHGB, CUL2, CXXC1, NSG1, DDC, DRD2, EGLN1, FBXO9, FGF13, GBE1, GPR37, GRIA3, HSPA4, NCOA1, NEFL, NFASC, NR4A2, NDF, NTRK2, OPA1, PAN2, PINK1, PPID, PRDX2, PTEN, RGS4, RTN1, SEPT5, SLC6A3, SLIT1, SPEN, SRSF7, SYNGR3, TCF7L2, TH, UBA1, UBB, UBE2I, UBE2K, UBE2L3, UCHL1, USP34, VAMP1, VDAC3, YWHAZ

The Human Parkinson’s Disease RT^2^ Profiler PCR Array was used to examine the effect of neuronal differentiation on the expression of PD-related genes in Diff control groups. Gene expression was analyzed on days 8 and 12 of neuronal differentiation. Of the 84 genes assessed, 57 were significantly upregulated or downregulated in at least one neural-differentiated group compared with the Undiff control group (Fig. 6). Among the differentially expressed genes, several are well established as critical regulators of dopaminergic neuron development, maintenance, and function. Brain-derived neurotrophic factor (BDNF) showed altered expression and is known to promote neuronal survival, differentiation, and synaptic plasticity, particularly supporting the viability and maturation of midbrain dopaminergic (mDA) neurons [26]. Further, the expression profile of significantly altered genes changes between day 8 and day 12 (Fig. 6A and B). PTEN-induced kinase 1 (PINK1), a key component of mitochondrial quality control, was also differentially expressed; PINK1 is essential for preserving mitochondrial integrity and protecting neurons from mitochondrial stress, a central feature of PD pathogenesis [27]. In addition, nuclear receptor subfamily 4 group A member 2 (NR4A2/Nurr1), a transcription factor required for dopaminergic neuron development and long-term survival, exhibited expression changes during neural differentiation. Nurr1 regulates genes involved in dopamine synthesis, vesicular transport, and neuronal maintenance [28]. Synaptogyrin 3 (SYNGR3), which is selectively enriched in dopaminergic synaptic vesicles, was also differentially expressed; this protein contributes to dopamine uptake and synaptic dopamine turnover, thereby influencing dopaminergic neurotransmission [29]. Furthermore, TH, the rate-limiting enzyme in dopamine biosynthesis and a hallmark of dopaminergic neuronal identity, showed differential expression consistent with ongoing neuronal differentiation [30].

Further enrichment analyses were performed to investigate a potential association between topo IIβ and neurodegeneration in the context of PD, with a focus on PD-associated genes. Based on the analyses conducted in this study, the difference in topo IIβ expression between the Diff control and α-Syn(A53T(+))/Diff group was most pronounced on day 12 of neuronal differentiation.

**Fig. 7:**
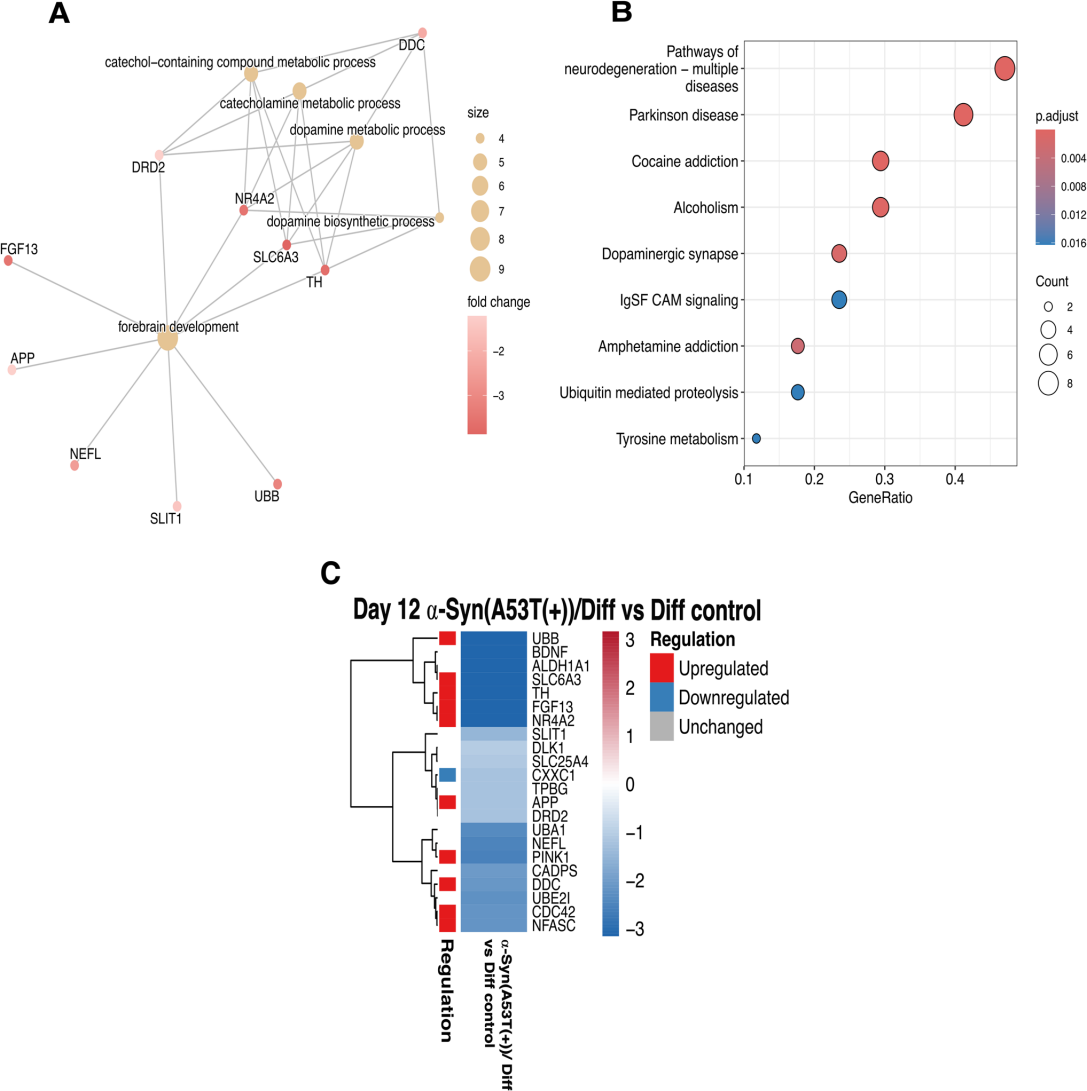
Enrichment analyses and heat map of Human Parkinson’s Disease RT^2^ Profiler PCR Array data. **A** cnetplot, **B** Kegg analyses, and **C** heat map displaying the difference in PD-associated gene expressions of day 12 α-Syn(A53T(+))/Diff group compared to day 12 Diff control. Genes that demonstrated a fold regulation (FR) < 0.5 were considered downregulated. The test group: α-Syn(A53T(+))/Diff and the control group: Diff control. The genes displayed in the heat map are listed as follows: ALDH1A1, APP, BDNF, CADPS, CDC42, CXXC1, DDC, DLK1, DRD2, FGF13, NEFL, NFASC, NR4A2, PINK1, SLC25A4, SLC6A3, SLIT1, TH, TPBG, UBA1, UBB, UBE2I

Accordingly, the gene expression array experiment was performed on day 12 of neuronal differentiation, using the α-Syn(A53T(+))/Diff group as the test group and the Diff control group for comparison, with the aim of identifying genes most significantly affected by α-Syn A53T(+). Among the 84 genes analyzed, 22 genes were found to be downregulated in the test group (Fig. 7C).

Pathway analyses of the significantly altered genes (Fig. 7 A and B) revealed the enrichment of several pathways closely associated with Parkinson’s disease, including neurodegeneration-multiple diseases, dopamine metabolism and biosynthesis, and the dopaminergic synapse. Within these pathways, several genes—such as SLC6A3 [31], NR4A2, and TH—are well established as key regulators of dopaminergic neuron function and dopamine biosynthesis.

Given that these pathways are widely recognized as PD-associated, their identification in the present analysis represents an important outcome of this study. Consistent with this, the pathways emerging from the genetic alterations induced in α-Syn(A53T(+))/Diff cells support the validity of this experimental system as an in vitro model of PD. Moreover, the involvement of these pathways reflects critical molecular mechanisms underlying dopaminergic dysfunction and disease-related neurodegeneration.

**Fig. 8:**
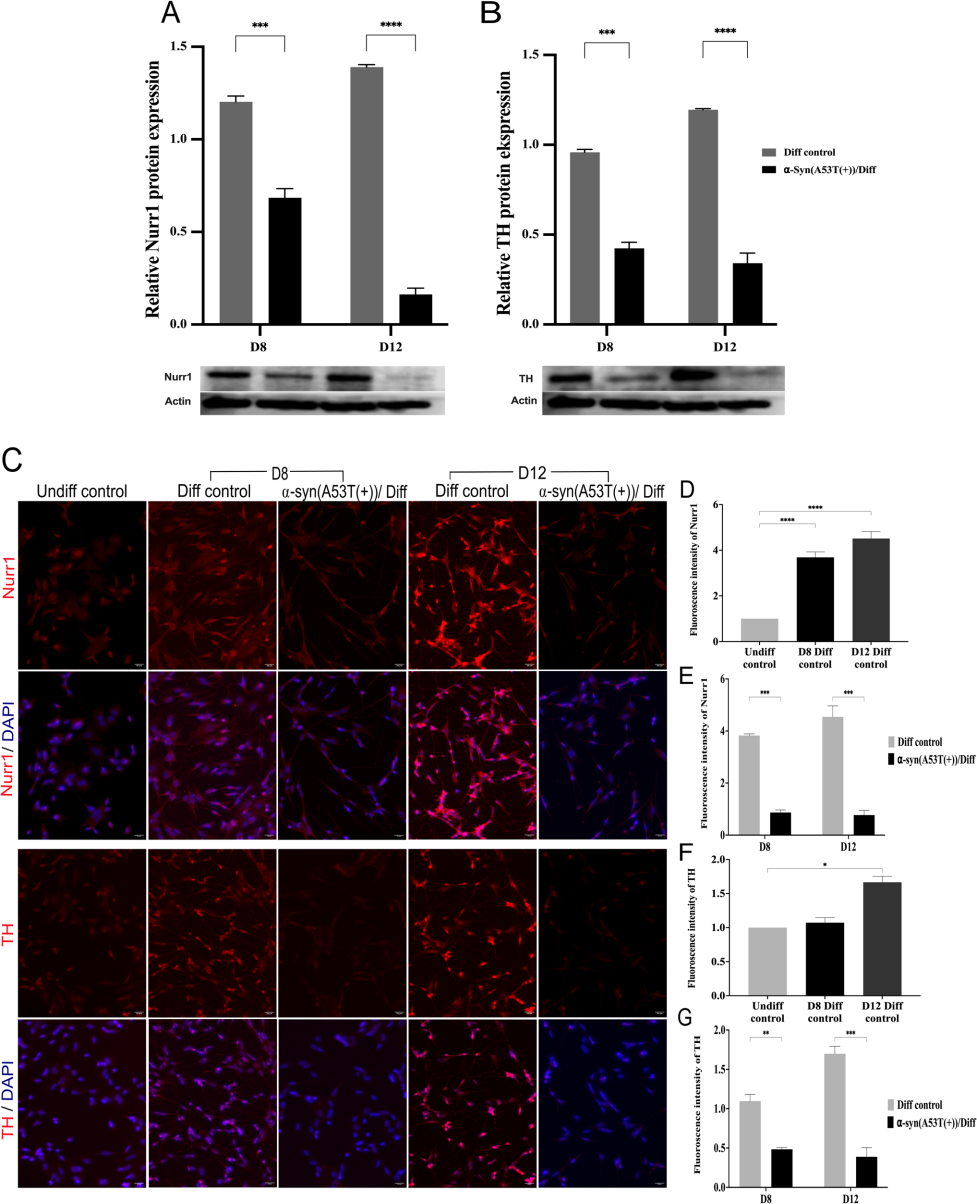
α-Syn A53T(+) decreased Nurr1 expression and dopaminergic neuronal biomarker TH in SH-SY5Y cells. Western blot analysis of **A** Nurr1, and **B** TH and IF staining of **C** Nurr1 and TH in Diff control and α-Syn(A53T(+))/Diff on the 8th and 12th day of neuronal differentiation. The images were taken under fluorescent microscope (20×). Neurite length analysis of **D** and **E** Nurr1 and **F** and **G** TH from IF staining images. Error bars represent the means ± standard deviation; for western blot, *n* = 3 samples in triplicate; *p < 0.05, **p < 0.01, ***p < 0.001, ****p < 0.0001. *Represents significance levels between groups

The protein level analysis of Nurr1 and TH was carried out to strengthen the potential connection of topo IIβ and PD. The Western blot result (Fig. 8A and B) revealed that both Nurr1 which has a role in development and maintenance of midbrain dopamine neurons [16] and TH which is known as a dopaminergic neuron biomarker and a specific enzyme in PD [32] are significantly upregulated in the Diff control group on day 8 and day 12 of neuronal differentiation. On the other hand, there was a significant gradual downregulation in the expression of Nurr1 and TH in the α-Syn(A53T(+))/Diff group, with the lowest levels (*p* < 0.0001) observed on day 12 of neuronal differentiation. The low expression of TH in this group provides evidence for the validity of the approach aimed at establishing a PD model. The IF staining findings (Fig. 8C) were consistent with the Western blot results, revealing a pronounced difference in fluorescence intensity between the Diff control and α-Syn(A53T(+))/Diff groups particularly on the 12th day of neuronal differentiation (*p* < 0.001). In addition, the network structure demonstrated by Nurr1 in neuronal differentiation demonstrates its contribution to neurogenesis in dopaminergic neurons [33]. Also, Nurr1 expression appeared to align with topo IIβ expression in the same groups across the same differentiation days, further supporting the potential positive feedback [15, 17] relationship between topo IIβ and Nurr1.

## Discussion

There has been a notable scientific focus on understanding the complex mechanisms involved in PD, especially the role of different molecular players in its pathology. This study specifically examines the role of topo IIβ in PD, with a particular emphasis on if α-Syn A53T(+) affects the expression of topo IIβ. Moreover, the study also involves analyzing the changes in dopaminergic markers, including Nurr1 and TH expression depending on α-Syn A53T(+). By using an in vitro PD model involving α-Syn A53T(+), the study aims to explore direct molecular relationships between topo IIβ expression and PD pathology development.

The increased levels of p-α-Syn observed in the PD model underscore its relevance to neurodegenerative disease pathogenesis [3]. Phosphorylation of α-Syn, particularly at Ser129, is widely recognized as a pathological modification associated with PD and is linked to early aggregation-prone states that may enhance aggregation propensity and contribute to neurotoxicity, rather than to fully mature fibrillar inclusions. In this study, immunofluorescence analysis using a pSer129-specific α-Syn antibody was employed to capture early molecular and cellular changes related to disease initiation and progression [34], rather than late-stage insoluble aggregates such as Lewy body–like inclusions. Consistent with this approach, the observed increase in pSer129 α-Syn immunoreactivity is interpreted as indicative of pathological α-Syn modification and increased aggregation propensity, rather than direct evidence of mature aggregate formation.

In the alignment with this early-stage focus, the A53T mutation was selected as a suitable model to investigate initial phases of α-Syn accumulation while minimizing confounding cellular dysfunction characteristic of later disease stages [7]. Accordingly, neuronal differentiation of SH-SY5Y cells was performed using a previously established protocol [20].

Neuronal differentiation was induced using RA and BDNF protocols in accordance with established literature, and the observed differentiation patterns were consistent with previously reported findings. Neuronal differentiation was successfully achieved by the 12th day, with morphological examinations showing cell enlargement and neurite development. The process of differentiation was accelerated from the 5th day of BDNF treatment, highlighting the neural differentiation-inducing effect of BDNF [20].

Our results indicate that α-Syn A53T(+) inhibits the process of neuronal differentiation in SH-SY5Y cells. Importantly, α-Syn(A53T(+))/Diff cells exhibited a significant delay in the acquisition of neural characteristics compared with Diff control cells. Moreover, neurite formation was significantly decreased, with fewer and shorter neurites, indicating poor development of neurons (Fig. 3). A reduced number of cells were observed, highlighting the cytotoxic properties of α-Syn A53T(+) aggregates. All experimental groups were initially seeded at identical cell densities. However, excessive production of α-Syn A53T(+) resulted in a selective reduction in cell number, indicating a harmful impact on neuronal development in SH-SY5Y cells. These observations are consistent with previous studies showing that the accumulation of α-Syn aggregates is associated with impaired neuronal function and cell death [3].

Moreover, the immunofluorescence labelling for MAP2 and NF-L demonstrated significant differences in the expression of neuronal markers between cells with and without overexpressed α-Syn in our study. While Diff control cells exhibited a steady increase in these markers, resulting in the formation of fully developed neural networks by day 12, α-Syn(A53T(+))/Diff cells displayed reduced expression levels. Specifically, the reduced levels of MAP2 and NF-L in cells overexpressing α-Syn A53T(+) emphasized the suppressive impact of mutant α-Syn on the development and structural stability of neurons [35].

Although α-Syn pathology is widely recognized as a central contributor to neuronal dysfunction in PD, there is still a considerable requirement to determine the specific molecular mechanisms involved in the sub-pathways impacted by the excessive production of α-Syn. The identification of these key molecules is crucial for better understanding of the mechanisms of PD and for potential therapy approaches. One potential candidate molecule in this particular scenario is the topo IIβ enzyme. Experimental studies have shown that the inhibition of this enzyme could be correlated with an upregulation of genes related to PD and AD [14, 18]. However, the disease stage at which topo IIβ dysfunction emerges, as well as the upstream factors contributing to its downregulation, remains largely unresolved.

Our study demonstrates that topo IIβ expression decreased at the protein level in the α-Syn(A53T(+))/Diff cells during neuronal differentiation, in contrast to Diff control cells. These findings suggest that pathogenic α-Syn accumulation is associated with suppressed topo IIβ expression under differentiation conditions.

Importantly, our observations are consistent with in vivo evidence reported by Kıyak et al., who demonstrated that early α-Syn pathology in A53T transgenic mice is accompanied by neuroinflammatory activation and a marked reduction in topo IIβ expression [19]. That study provided independent support for the notion that topo IIβ downregulation is an early molecular event associated with α-Syn pathology, occurring alongside inflammatory responses rather than as a late consequence of neuronal loss. Together, these findings from both in vitro and in vivo A53T models strengthen the view that decreased topo IIβ expression represents an early and disease-relevant molecular alteration linked to pathogenic α-Syn burden.

Nevertheless, the present study was not designed to establish direct mechanistic causality between topo IIβ suppression and dopaminergic impairment. As such, our data do not allow us to distinguish whether reduced topo IIβ expression acts as a driver of impaired differentiation or arises as a downstream consequence of α-Syn-induced cellular stress and toxicity. Functional rescue experiments, including gain-of-function and loss-of-function approaches targeting topo IIβ, will be required to address this question and are therefore an important direction for future investigations. Accordingly, our conclusions are framed to emphasize molecular association rather than direct regulation.

Beyond topo IIβ, we examined broader transcriptional changes associated with neuronal differentiation and α-Syn A53T(+) on the expression of PD-associated genes using the Human Parkinson’s Disease RT^2^ Profiler PCR Array. Neuronal differentiation alone markedly changed gene expression, with 57 of 84 genes exhibiting differential regulation on days 8 and 12 relative to Undiff control. These changes reflect the activation of neurodevelopmental and neuroprotective pathways, crucial for the maturation of dopaminergic neurons. Genes including BDNF, PINK1, NR4A2 (Nurr1), SYNGR3, and TH were significantly elevated, underscoring the attainment of a dopaminergic identity. In the α-Syn(A53T(+))/Diff group, a notable change occurred: 22 Parkinson’s disease–related genes were markedly downregulated, including TH, NR4A2, BDNF, and PINK1. This pattern indicates that pathogenic α-Syn disrupts neuroprotective and differentiation-associated processes severely. Four of the downregulated genes—aldehyde dehydrogenase family 1, subfamily A1 (ALDH1A1 (Ahd2)), an enzyme participating in retinoic acid biosynthesis [36], TH, solute carrier family 6 (neurotransmitter transporter, dopamine), member 3 (SLC6A3), which is a dopamine transporter [31], delta-like 1 homolog (DLK1), which encodes a transmembrane protein with numerous epidermal growth factor repeats that regulates cell development [37], and BDNF have previously been reported as downstream targets of Nurr1 [26, 38–41] (Fig. 7). These genes have been identified as the key regulators of dopaminergic neuronal differentiation, cell survival, and dopamine synthesis [42, 43]. Rather than interpreting these changes as independent events, our findings suggest that α-Syn-associated suppression of dopaminergic gene networks may converge on shared regulatory pathways. However, we emphasize that our data demonstrate coordinated downregulation, not direct transcriptional control by Nurr1 or topo IIβ within this experimental framework.

The potential relationship between Nurr1 and topo IIβ has been proposed in prior studies. Nurr1 has been reported to influence topo IIβ expression [17], while topo IIβ activity has been implicated in regulating transcription of neuronal genes, including Nurr1 itself [15]. These observations raise the possibility of a bidirectional regulatory interaction. In the present study, the parallel reduction of topo IIβ and Nurr1 expression in the α-Syn A53T(+) model is consistent with this concept but does not provide direct functional evidence of a feedback loop.

**Fig. 9:**
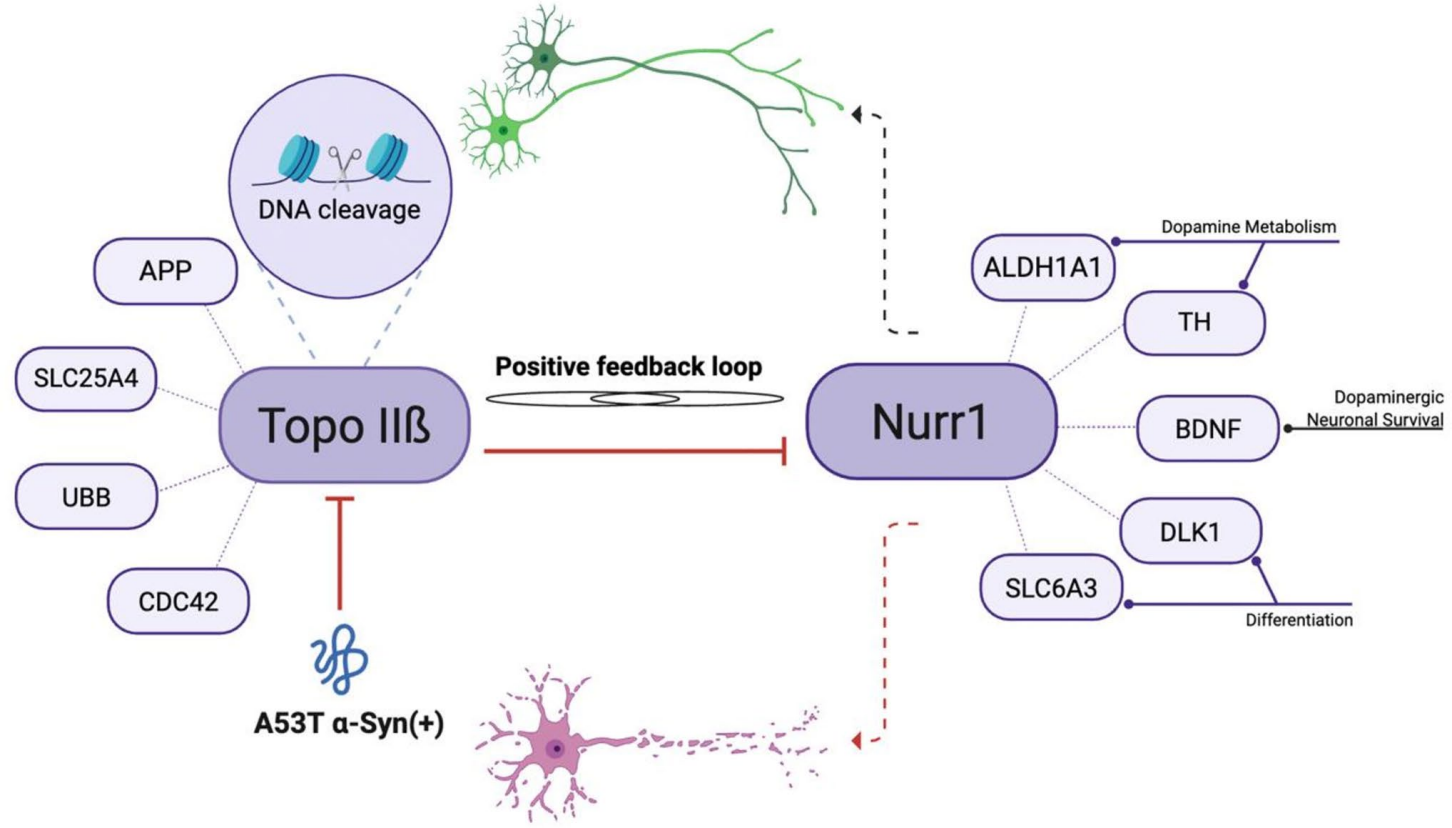
Hypothetical model illustrating a potential positive feedback mechanism between topo IIβ and Nurr1. Topo IIβ may be involved in the expression of PD-related genes that are targets of Nurr1, and both have been reported to act as each other’s downstream targets [15] [17], suggesting a mutual regulatory relationship

To determine whether our results of gene expression obtained from the RT^2^ Profiler PCR Array are dependent on the suppression of topo IIβ, the data was compared with recent studies that investigated genes affected during neuronal differentiation following selective silencing of topo IIβ. The data was compared with the microarray study conducted in 2015 investigating the gene expression changes related to neurodegenerative diseases following silencing of topo IIβ during neural differentiation of human mesenchymal stem cells (hMSCs) [14] which revealed the following genes—amyloid precursor protein (APP), involved in synaptogenesis and synaptic plasticity [44], mitochondrial ATP/ADP translocator (SLC25A4), inner mitochondrial membrane transporter [45], and ubiquitin B (UBB), ubiquitin encoding gene [46] as well as with the study that explored the downregulation of Nurr1 and Cdc42, a Rho GTPase family member, also due to topo IIβ silencing [15]. Considering that topo IIβ has also been proposed as a downstream target of Nurr1, this bidirectional relationship implies that their interaction may significantly influence molecular pathways related to PD (Fig. 9). Therefore, the model presented in Fig. 9 should be regarded as hypothetical, integrating previous literature with correlative expression changes observed in this study and in A53T mouse models, including the work by Kıyak et al. Further mechanistic studies will be required to determine whether such interdependence operates under pathological conditions.

To corroborate the array results, we additionally evaluated TH and Nurr1 expression levels via RT-qPCR and Western blot analysis. The downregulation of TH in our PD model is significant, as it reflects the progressive loss of dopaminergic neurons seen in PD patients. Given that TH is the rate-limiting enzyme in dopamine production, its diminished expression likely contributes to the dopaminergic deficit typical of the condition. Conversely, TH levels exhibited a normal increase throughout neural differentiation, affirming a coherent and functional dopaminergic maturation process. The significant reduction in TH expression in the α-Syn(A53T(+))/Diff group underscores the pathogenic effect of α-Syn A53T(+) on the integrity of dopaminergic neurons.

The PCR array analysis at day 12 of neuronal differentiation revealed distinct transcriptional changes in α-Syn(A53T(+))/Diff that align with PD pathology. The identification of downregulated genes involved in the ubiquitin-proteasome system (UBB, UBE2L, UBA1) is particularly interesting, as impaired protein degradation is recently linked to α-Syn-mediated neurotoxicity in PD [47, 48]. The cnetplot analysis revealed “forebrain development” as a central regulatory hub connecting multiple downregulated genes including APP, FGF13, NR4A2, and TH, suggesting that α-Syn A53T(+) may disrupt normal neurodevelopmental pathways that persist in mature dopaminergic neurons. The significant enrichment of neurodegeneration-related pathways and dopaminergic signaling, coupled with the upregulation of key dopaminergic markers such as TH, SLC6A3, NR4A2, and DDC [49], provides strong molecular validation for the α-Syn(A53T(+))/Diff system as a relevant in vitro model of PD. Furthermore, the differential expression pattern observed in the heatmap demonstrates clear segregation between control and α-Syn(A53T(+))/Diff, with genes such as DRD2 and NEFL showing marked downregulation, potentially indicating compromised dopamine receptor signaling and neurofilament organization [50, 51]. These transcriptional changes collectively recapitulate key molecular features of PD pathogenesis and underscore the utility of this model for investigating the mechanistic links between α-Syn toxicity, dopaminergic dysfunction, and neurodegeneration.

Taken together, our findings are consistent with a model in which early α-Syn-associated changes are accompanied by coordinated suppression in topo IIβ, Nurr1, and key dopaminergic genes, paralleling patterns reported in A53T transgenic mice. While these results do not establish direct regulatory or causal relationships, they identify topo IIβ as a molecularly vulnerable node within α-Syn-associated transcriptional dysregulation. Elucidating the functional significance of this association will require future studies incorporating targeted manipulation of topo IIβ and Nurr1, as well as integration of neuroinflammatory mechanisms implicated in early α-Syn pathology.

## Limitations and Future Directions

This study has several limitations. Although appropriate transfection controls are important for in vitro models, the primary aim of this work was to examine molecular changes associated with α-Syn A53T(+) as a PD-related stress model rather than to comprehensively assess transfection-related effects. While EGFP is widely used as a transfection marker in SH-SY5Y cells and is generally not reported to markedly affect neuronal differentiation or viability under comparable conditions [52], the absence of an EGFP-only control represents a limitation of this study. In addition, the lack of direct comparison with wild-type α-Syn limits conclusions regarding mutation-specific effects. Furthermore, although RA/BDNF-differentiated SH-SY5Y cells are commonly used as a dopaminergic neuron–like model, they do not fully recapitulate mature dopaminergic neuron identity, synaptic architecture, or functional properties, and differentiation efficiency may vary depending on the protocol [53]. Therefore, the present findings should be interpreted within the constraints of this cellular model, and future studies using more physiologically relevant systems will be required to validate these observations.

## Conclusion

In conclusion, this study shows that α-Syn A53T (+) is associated with marked alterations in the expression of key dopaminergic neuronal markers during in vitro neuronal differentiation in SH-SY5Y cells. The observed downregulation of topo IIβ, Nurr1, and TH, together with altered neuronal morphology and differentiation patterns, indicates that pathogenic α-Syn expression is accompanied by changes in transcriptional programs associated with dopaminergic neuron identity and maintenance. While the present findings do not establish a direct causal relationship, they highlight potential molecular associations through which early α-Syn-related pathology may contribute to dopaminergic vulnerability in PD. These results provide a framework for future studies aimed at functionally validating the roles of topo IIβ and Nurr1 and exploring therapeutic strategies that target early transcriptional dysregulation in PD.

## Supplementary Information

Below is the link to the electronic supplementary material.

ESM 1 (PNG 2.05 MB)

ESM 1 Effect of initial seeding density on neuronal differentiation. To assess whether differences in cell number influence neuronal differentiation outcomes, the initial seeding density was varied by ± 20% relative to the standard condition. Representative images show the progression of neuronal differentiation under reduced (− 20%) and increased (+ 20%) seeding densities. No evident differences in differentiation efficiency were observed across conditions (TIF 32.5 MB)

## Data Availability

Data sharing not applicable to this article as no datasets were generated or analyzed during the current study.
