# Supplementary figures and images for: A53T α-Synuclein Expression is Associated with Altered Dopaminergic-Like Differentiation and Reduced DNA Topoisomerase IIβ Levels in an In Vitro Model of Parkinson’s Disease
Source: Mol Neurobiol. 2026 May 11;63(1):618. doi: 10.1007/s12035-026-05881-1 (PMC13161279; doi:10.1007/s12035-026-05881-1)

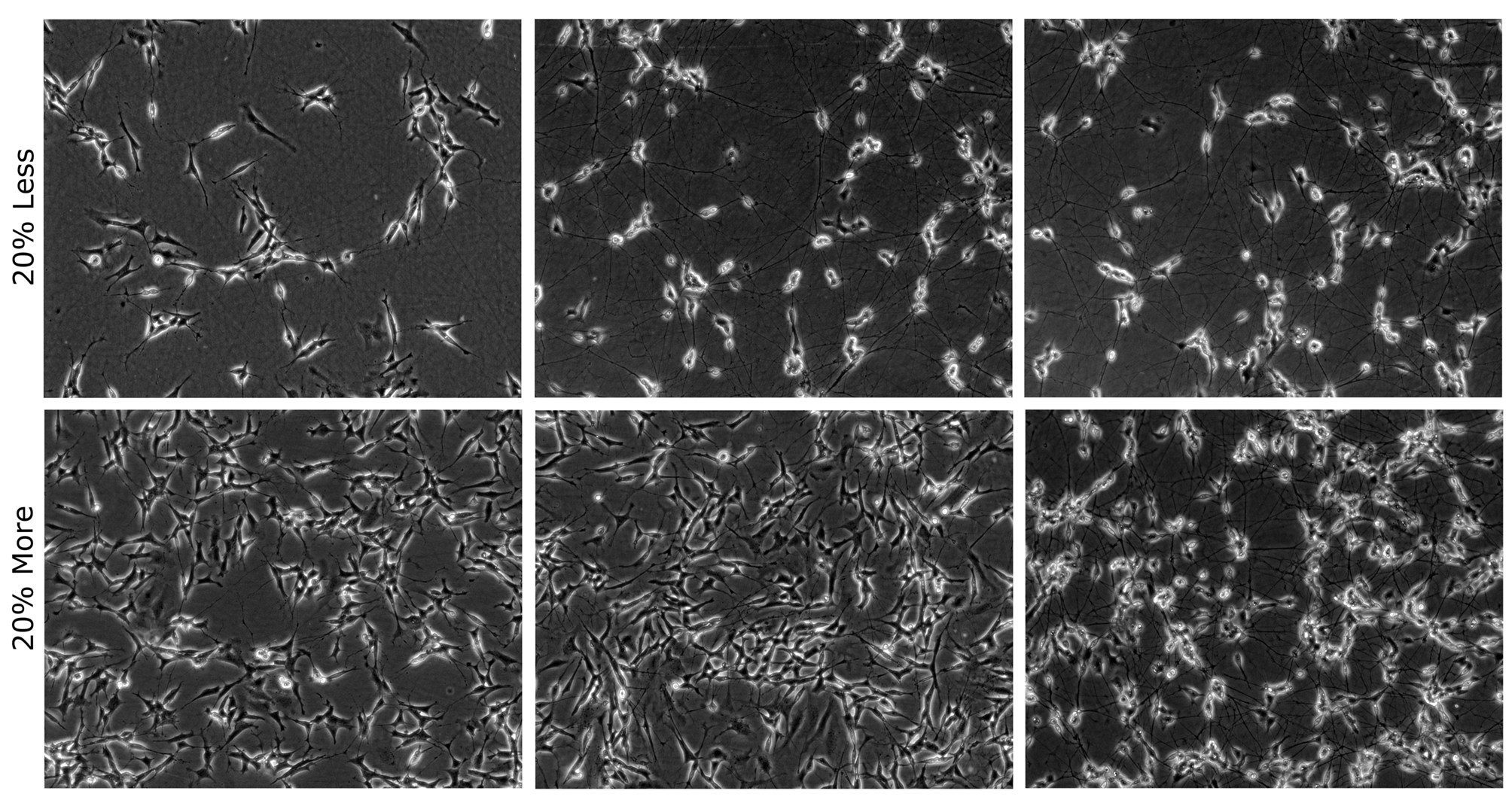

Supplement: Supplementary file 1 — (PNG 2.05 MB) [file 12035_2026_5881_Fig10_ESM.png]

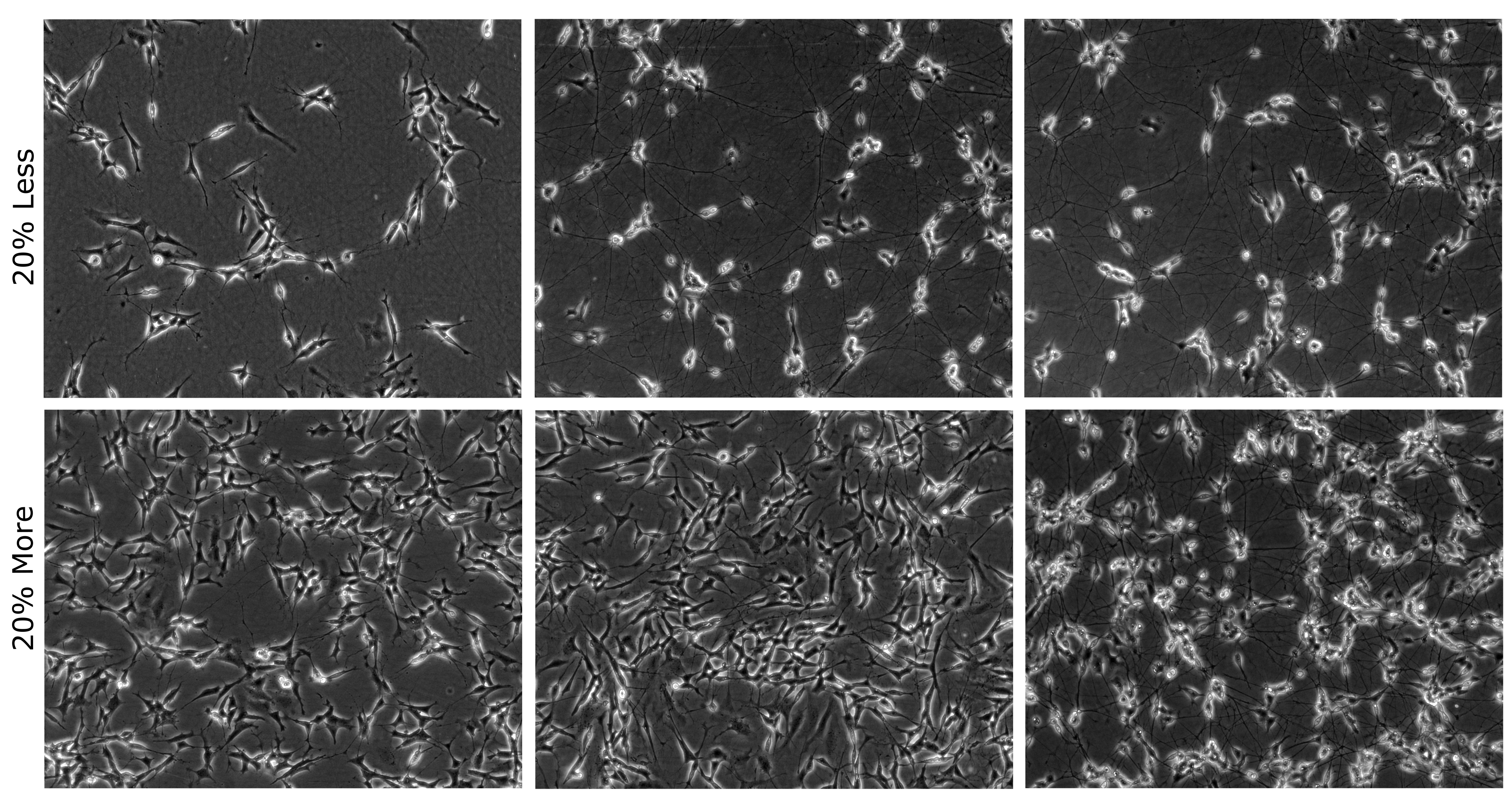

Supplement: Supplementary file 2 — Effect of initial seeding density on neuronal differentiation. To assess whether differences in cell number influence neuronal differentiation outcomes, the initial seeding density was varied by ± 20% relative to the standard condition. Representative images show the progression of neuronal differentiation under reduced (− 20%) and increased (+ 20%) seeding densities. No evident differences in differentiation efficiency were observed across conditions (TIF 32.5 MB) [file 12035_2026_5881_MOESM1_ESM.tiff]
